# An Innovative Technique for the Repair of Long-Segment Juxtaductal Coarctation of the Aorta in Neonates and Infants

**DOI:** 10.7759/cureus.99051

**Published:** 2025-12-12

**Authors:** Rupesh Kumar, Arun Sharma, Sanjeev H Naganur, Vendra S R, Jigyasa Bharti

**Affiliations:** 1 Cardiothoracic and Vascular Surgery, Postgraduate Institute of Medical Education and Research, Chandigarh, IND; 2 Radiology, Postgraduate Institute of Medical Education and Research, Chandigarh, IND; 3 Cardiology, Postgraduate Institute of Medical Education and Research, Chandigarh, IND; 4 Cardiothoracic and Vascular Anesthesiology, Postgraduate Institute of Medical Education and Research, Chandigarh, IND

**Keywords:** complex coarctation, end-to-end vascular anastomosis, juxtaductal aorta, left subclavian flap aortoplasty, severe coarctation of the aorta

## Abstract

An extra-cardiac anomaly known as coarctation of the aorta (CoA) is characterized by intraluminal constriction that impedes forward blood flow. The most typical location is close to the ductus arteriosus, immediately distal to the left subclavian artery. Blood flow obstruction is typically caused by a "shelf-like" tissue that protrudes from the posterior aortic wall into the aortic lumen. Long-segment juxtaductal CoA with cardiac dysfunction is a surgical priority. End-to-end anastomosis of long-segment juxtaductal CoA leads to a considerable gradient across the segment even after extensive mobilization. Addition of a subclavian flap along with the end-to-end anastomosis of the posterior layer of the aorta is an achievable, physiologic, and feasible procedure.

## Introduction

Coarctation of the aorta (CoA) is an extra-cardiac abnormality characterized by intraluminal constriction that impedes forward blood flow. Just distal to the left subclavian artery, close to the ductus arteriosus region, is the most frequently observed location [1]. A "shelf-like" tissue that protrudes from the posterior aortic wall into the aortic lumen is typically the cause of blood flow obstruction.

For coarctation repair, a number of surgical techniques have been established, including patch aortoplasty, prolonged end-to-end anastomosis, subclavian flap angioplasty, resection and end-to-end anastomosis, and tubular bypass grafts [2]. The only methods that completely remove the coarcted shelf segment in these steps are those that involve resection and either end-to-end anastomosis or extended end-to-end anastomosis. In contrast, there is a substantial risk of recoarctation, and the native coarcted section remains in place after other surgical procedures.

If the coarcted segment is long, a typical end-to-end anastomosis becomes difficult because aortic mobility over a long distance increases the risk of airway and/or anastomotic blockage due to the kinking of the proximal aorta caused by the overstretching of the aortic lumen. Additionally, stress on the anastomosis site might result in a potentially fatal blowout that causes a significant hemorrhage. There are several widely recognized techniques for reducing stress, such as subclavian flap aortoplasty. The shelf-like or membranous curtain-like structure in the aortic segment remains intact after a traditional subclavian flap aortoplasty. As a result, while an immediate reduction in the pressure gradient across the segments may be accomplished, these patients eventually experience severe recoarctation, making repeat surgeries extremely difficult [3].

We will discuss the surgical strategy in one of our cases of CoA in a two-month-old child who presented to us with symptoms of heart failure and whose computed tomography (CT) aortogram revealed a long-segment juxtaductal CoA which is also known as infantile coarctation or tubular hypoplasia of a segment of the aorta. Our procedure involved taking advantage of both the end-to-end and subclavian flap procedures. The complication of limb hypoperfusion or growth can be dealt with through regular limb exercises.

## Case presentation

Surgical technique

**Figure 1:**
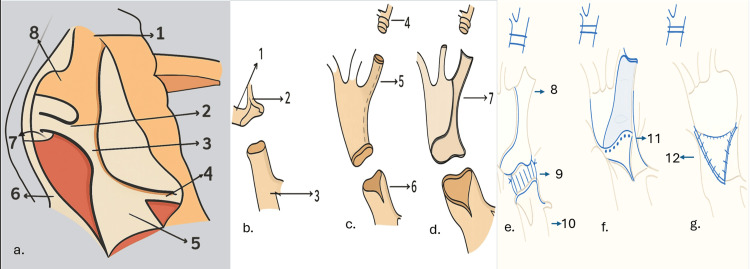
Schematic line diagram showing the juxtaductal coarctation of the aorta and the operative steps (a) The anatomy of juxtaductal coarctation of the aorta exposed through left posterolateral thoracotomy (1: left subclavian artery; 2: patent ductus arteriosus; 3: coarcted segment of the juxtaductal aorta; 4: intercostal artery; 5: descending thoracic aorta; 6: left vagus nerve; 7: left recurrent laryngeal nerve; 8: distal aortic arch). (b) Resection of the coarcted segment of the aorta (1: distal aortic arch; 2: left subclavian artery; 3: descending thoracic aorta). (c) Left subclavian longitudinal arteriotomy (4: distal end of the left subclavian artery clipped; 5: left subclavian longitudinal arteriotomy; 6: descending thoracic longitudinal aortotomy for a few millimeters). (d) Flap of the left subclavian artery (7: flap of the left subclavian artery). (e) Anastomosis of the posterior layer of the descending thoracic aorta with the distal aortic arch (8: subclavian artery flap; 9: suture anastomosis between the posterior layer of the distal aortic arch with the posterior wall of the descending thoracic aorta; 10: descending thoracic aorta). (f) Completion of the anastomosis of the posterior layer of the descending thoracic aorta with the posterior wall of the distal aortic arch (11: suture anastomosis of the posterior layer of the descending thoracic aorta with the posterior wall of the distal aortic arch completed). (g) Aorta after left subclavian aortoplasty (12: suture anastomosis of the left subclavian artery flap with the anterior wall of the descending thoracic aorta). Image Credit: Authors (using BioRender and Keynote)

The procedure is performed through a left lateral thoracotomy. An arterial monitoring line is placed in the right radial artery. An additional arterial monitoring line is placed on a lower extremity. A pulse oximetry monitor is placed on a lower extremity as well. The patient is placed in the right lateral decubitus position and prepped for a left lateral, muscle-sparing thoracotomy. The chest cavity is entered through the fourth intercostal space, the lung is retracted anteriorly, and the mediastinal pleura is opened over the area of coarctation and retracted with stay sutures. The vagus and left recurrent laryngeal nerves are then identified (Figure 1a). The ductus arteriosus can be encircled and securely tied just before or after the vascular clamps are placed on the proximal and distal aorta. Proximal control is obtained with a C-clamp that is applied to include the distal aortic arch, whereas the left subclavian artery is mobilized till distally and is encircled with a suture. Distal control is obtained with a Satinsky-type clamp. Systemic heparinization with a dose of 1 mg/kg of unfractionated heparin is administered at least three minutes before the clamping of the aorta. After the clamps have been placed and the ductus has been ligated, the distal end of the coarcted segment of the aorta is incised transversely, and it is split anteriorly for a few millimeters (Figure 1b), followed by the segment of the aorta proximal to the coarctation. Now, the left subclavian artery longitudinal arteriotomy is performed (Figure 1c) starting from the cut end of the aorta extending just before the origin of the vertebral artery (Figure 1d).

The posterior anastomosis is performed between the two ends of the aorta with an appropriate suture (Figure 1e). We prefer a 6-0 delayed absorbable polydioxanone suture for posterior wall anastomosis. After the posterior anastomosis is completed (Figure 1f), the anterior deficient lumen is anastomosed with the turned-down subclavian artery flap with a separate 6-0 delayed absorbable polydioxanone suture (Figure 1g). The ends of the sutures are tied to each other. The advantage of this technique is that the coarcted segment of the aorta with its shelf is excised out, as well as the subclavian flap tension-free anastomosis is also achieved, thereby allaying the risk of left-over coarcted segment and unnecessary tension and kinking of the proximal aorta after anastomosis. At the conclusion of the repair, the distal clamp is removed first to allow for deairing and initial inspection of the suture line. There may be an initial gradient between the upper and lower extremity blood pressures due to lower extremity vasoconstriction or to a Coanda effect that favors flow up of the innominate artery as opposed to around the transverse aortic arch; however, this should resolve within a few minutes. The accepted blood pressure gradient in our setup is less than 9 mmHg.

**Figure 2:**
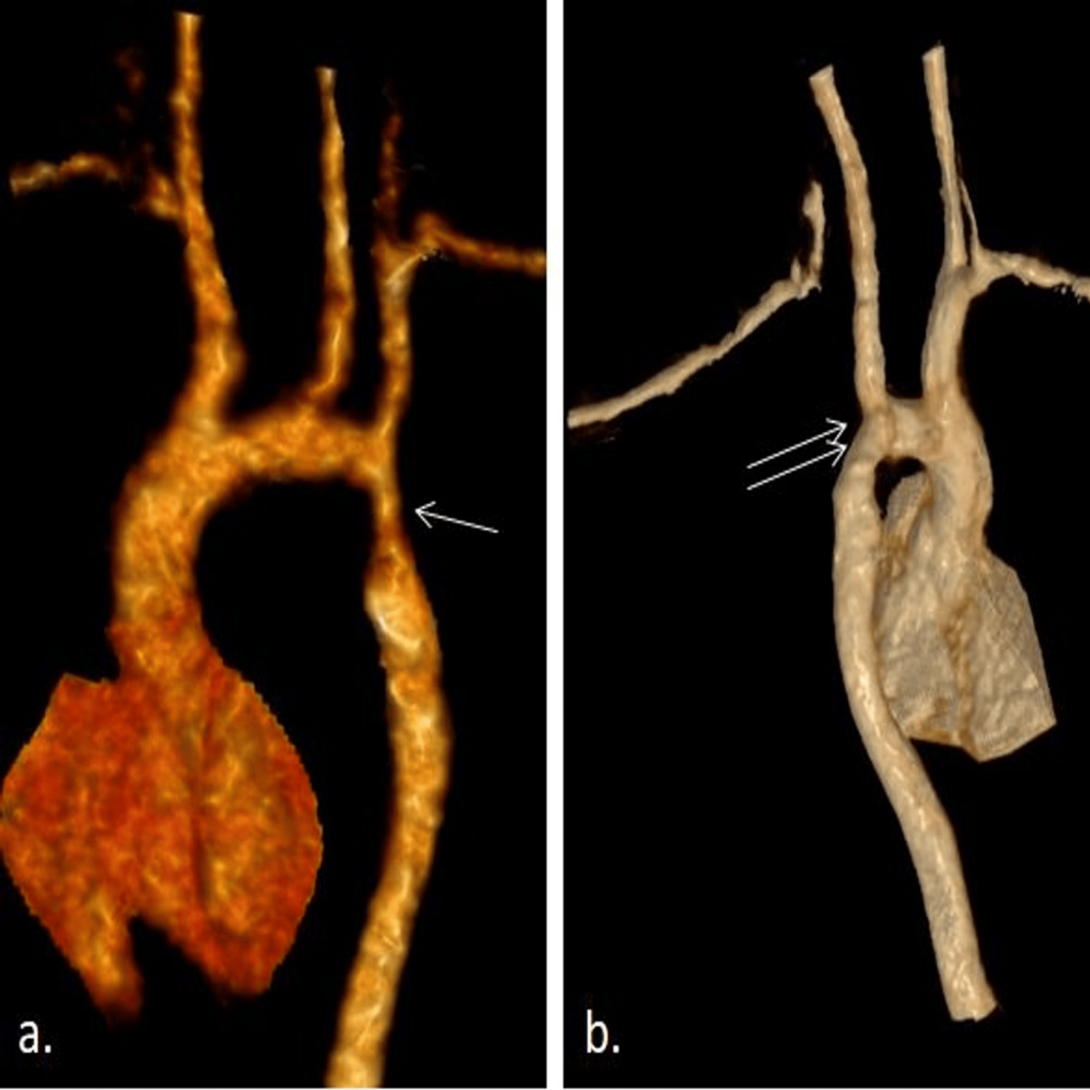
(a) Preoperative CT imaging showing the long-segment coarctation of the aorta (single arrow). (b) Postoperative CT imaging one year after the repair of coarctation of the aorta showing normal luminal continuity (double arrow) CT: computed tomography Image Credit: Authors (using BioRender and Keynote)

The follow-up of one of our infants at 12 months after surgery revealed an asymptomatic child with normal tone, power, and growth of the left upper extremity. Two-dimensional echocardiogram revealed a systolic gradient of 3 mmHg across the anastomosis and no evidence of recoarctation and normal growth of the distal aorta. The CT aortogram also showed a significant growth of the luminal dimension of the aorta without any recoarctation of the segment of anastomosis (Figure 2).

A follow-up study of patients undergoing CoA repair has documented approximately a 10% incidence of recoarctation 10 years following surgery. Around 79% were patients who underwent resection and end-to-end anastomosis, and 10% had extended resection and end-to-end anastomosis, while the remaining 11% patients underwent subclavian flap angioplasty and total arch reconstruction, respectively, and developed recoarctation [4].

Subclavian flap anastomosis is preferred for long-segment coarctation repair as it does not lead to the extensive mobilization of the aorta; hence, it is advantageous as there is no immediate gradient across the anastomosis. However, as the pathological coarcted segment is still persisting in the native aorta, in the long term, it will again lead to a significant gradient and recoarctation. The concern of left upper limb ischemia following left subclavian artery transection is overimagined as extensive collateralization for the upper limb perfusion from the left vertebral artery and intercostals leads to the normal growth and power of the upper extremity muscles [4].

## Discussion

About 10% of patients who had CoA repair experience recoarctation 10 years after surgery, according to a follow-up study [4]. Ten percent had extended resection and end-to-end anastomosis, while the remaining 11% had subclavian flap angioplasty and entire arch repair, respectively, and 79% had resection and end-to-end anastomosis. Because it does not result in extensive aortic mobilization, subclavian flap anastomosis is the preferred method for repairing long-segment coarctation. The procedure is advantageous because there is no gradient across the anastomosis right away, but because the pathological coarcted segment is still present in the native aorta, it will eventually cause a significant gradient and recoarctation. Since the left vertebral artery and intercostals provide significant collateralization for the upper limb circulation, which results in the normal growth and power of the upper extremity muscles, the risk of left upper limb ischemia after left subclavian artery transection is overestimated.

Preference for a specific surgical technique, aortic juxtaductal coarctation, is influenced by the lesion's form and extent, the existence of ductus arteriosus and related abnormalities, and age at presentation. The goal of any surgical procedure is to avoid creating any gradient across the restored aortic segment. Objective comparisons can be established based on factors such as the rate of recoarctation and freedom from re-intervention. Long-segment coarctation is best treated by prolonged end-to-end anastomosis, while resection of the coarcted segment with end-to-end anastomosis is a straightforward and optimal technique for discrete lesions. The risk of long-segment coarctation is that a few intercostal branches may be sacrificed during mobilization. Additionally, prolonged mobilization may cause the proximal aorta to unintentionally kink, creating a gradient across the repaired segment. Additionally, the left main bronchus experiences extrinsic compression due to significant mobilization and anastomosis, which compromises airways. Since subclavian flap anastomosis does not result in extensive aortic mobilization, it is the preferred method for repairing long-segment coarctation. The procedure is advantageous because there isn't an immediate gradient across the anastomosis; however, because the pathological coarcted segment is still present in the native aorta, it will eventually result in a significant gradient. After we looked more closely at the specifics of these operations, we came up with a method that would maximize benefits and eliminate the chance of recurrence by utilizing the advantages of all the methods mentioned above [5].

CoA in newborns and young children can be surgically corrected by left subclavian artery flap aortoplasty, especially when the stenosis includes an extended aortic segment or is linked to arch hypoplasia [6]. To provide a seamless, tension-free repair, the transacted subclavian artery is used as a pedicled flap to expand the constricted aortic lumen. In appropriate pediatric patients, the use of native tissue offers the possibility of somatic growth and better physiological and long-lasting repair, resulting in a long-lasting solution. This approach avoids potential issues with prosthetic material by using native tissue [7].

In certain instances, the constricted aortic segment is long and is widened by patch aortoplasty and the left subclavian artery flap aortoplasty. The primary goal is to prevent future recoarctation and expand the constricted segment. All things considered, left subclavian artery flap aortoplasty is regarded as a dependable and successful surgical option, especially for newborns and infants with distinctive CoA anatomy. It exhibits a low rate of recoarctation and good long-term results [8]. The main compromise, though, is the sacrifice of the left subclavian artery, which usually lowers left arm blood pressure and pulse strength. A slight reduction in forearm growth variables could lead to clinically relevant problems. Long-term left arm morbidity and ischemia are additional risks associated with this treatment. When compared to the right arm, this condition frequently shows up as quantifiable changes in the affected limb, such as slight decreases in forearm muscle mass, blood pressure, grip strength, and general size (hypoplasia) [8].

Therefore, we employ the method of end-to-end posterior layer anastomosis in conjunction with anterior layer subclavian flap anastomosis in our case.

## Conclusions

This innovative technique of addition of a subclavian flap along with the end-to-end anastomosis of the posterior layer of the aorta is a much more viable, physiologic, and reproducible procedure and can be a viable surgical option for the repair of long-segment coarctation in infants. It involves the advantage of a partial end-to-end anastomosis with a left subclavian flap to cover the anterior defect, thereby achieving a fully expandable lumen of the aorta without much difficulty, and hence should be a favored procedure over an interposition tube graft.
